# Serological, Molecular, and Epidemiological Investigation of *Toxoplasma gondii* Infection in Blood Donors from the Brazilian Semiarid Region

**DOI:** 10.3390/tropicalmed11060163

**Published:** 2026-06-17

**Authors:** Basílio Felizardo Lima Neto, Ana Caroline Dantas Amorim, Maria Jessianny Diniz Alves, Ana Maria Santos Lima, Janielton Albuquerque Lima, Celine Sousa Menezes Sá, Emilly Henrique Silva, João Luís Garcia, Vinicius Longo Ribeiro Vilela, Thais Ferreira Feitosa

**Affiliations:** 1 Postgraduate Program in Science and Animal Health, Federal University of Campina Grande—UFCG, Patos 58055-018, PB, Brazil; felizardobasilio95@gmail.com (B.F.L.N.); carolinedantasvet@gmail.com (A.C.D.A.); vinicius.vilela@ifpb.edu.br (V.L.R.V.); 2 Department of Veterinary Medicine, Federal Institute of Paraíba—IFPB, Sousa 58805-345, PB, Brazil; jessianny.diniz@academico.ifpb.edu.br (M.J.D.A.); analimaal372@gmail.com (A.M.S.L.); janielton.albuquerque@academico.ifpb.edu.br (J.A.L.); celine.sousa@academico.ifpb.edu.br (C.S.M.S.); henrique.emilly@academico.ifpb.edu.br (E.H.S.); 3 Department of Preventive Veterinary Medicine, State University of Londrina—UEL, Londrina 86057-970, PR, Brazil; incttoxo@uel.br

**Keywords:** blood transfusion, epidemiology, immunocompromised individuals, PCR, serology, toxoplasmosis

## Abstract

This study aimed to determine the prevalence and risk factors associated with *Toxoplasma gondii* infection in blood donors from the Brazilian Semiarid region, and to explore its implications for transfusion safety. Samples were collected from 646 donors at blood donation centers in the states of Ceará and Paraíba. Serological diagnosis was performed using BIOLISA TOXOPLASMOSE ELISA kits for anti-*T. gondii* IgM and IgG antibodies, and molecular diagnosis was conducted by conventional PCR targeting a 529-bp noncoding repetitive fragment. Epidemiological questionnaires on variables associated with infection were administered, and statistical analysis was performed in univariate and multivariate stages, using multiple logistic regression. Among the 646 donors, 43.4% (281/646) were positive for anti-*T. gondii* IgG antibodies, 0.3% (2/646) for IgM antibodies, and none tested positive by PCR. In the univariate analysis, age, family income, educational level, salad washing practices, water source, raw milk consumption, and duration as a donor were significantly associated, whereas in the multivariate analysis only “age” and “salad washing practices” remained significant. A substantial IgG seroprevalence was observed among blood donors in the Brazilian Semiarid. The low IgM frequency, concurrent IgG positivity, and negative PCR results are consistent with a low transfusion risk in the region. However, these findings should be interpreted cautiously, as negative PCR results do not completely rule out the presence of circulating parasites. Age was identified as a risk factor, whereas proper salad washing showed a protective effect.

## 1. Introduction

*Toxoplasma gondii* is an obligate intracellular protozoan whose definitive hosts are felids, which contaminate the environment by shedding oocysts in their feces. It is the etiological agent of toxoplasmosis, a cosmopolitan zoonosis [1]. Toxoplasmosis has notable relevance in the Brazilian Semiarid region, where seroprevalence rates of up to 66% have been reported in humans [2]. This region presents specific environmental characteristics that may influence the dynamics of the *T. gondii* life cycle [3].

Under semiarid conditions, oocyst sporulation may occur within 12 h after fecal deposition during dry season [3], allowing the parasite to become infective rapidly and increasing the likelihood of infection when infected felids defecate on animal pastures [4]. Animals destined for slaughter may acquire the infection and transmit it to humans through consumption of raw or undercooked meat [5,6].

In humans, infection is generally foodborne, resulting from ingestion of tissue cysts in undercooked meat or sporulated oocysts in contaminated water or vegetables [7]. Nevertheless, transmission may also occur through blood transfusion when infected donors in parasitemic phases transmit *T. gondii* to recipients via tachyzoites present within immune cells or free in the bloodstream. Cases of transfusion-transmitted toxoplasmosis have been documented, particularly following transfusion of leukocyte-rich blood products to immunocompromised recipients, highlighting the clinical relevance of this transmission route [8,9]. An infected blood unit may represent a significant risk, particularly for immunocompromised individuals [10], as immunocompetent persons are usually asymptomatic [11]. In contrast, immunocompromised patients may develop severe manifestations such as encephalitis, chorioretinitis, pneumonia, meningitis, fetal malformations, and abortion [12,13].

In this context, transfusion safety is a relevant concern in blood donation systems worldwide, with a global prevalence of 33% reported for *T. gondii*. This is even more evident in Brazil, where the highest prevalence among blood donors has been observed, with 75% positivity for *T. gondii* [14]. Nevertheless, routine laboratory screening in Brazilian blood centers does not include diagnostic testing for *T. gondii* [15]. Several serological methods are available for antibody detection, among which the enzyme-linked immunosorbent assay (ELISA) stands out due to its high sensitivity and specificity [16]. For molecular detection, polymerase chain reaction (PCR) demonstrates approximately 100% sensitivity and specificity [17], and the human buffy coat may be used for molecular diagnosis in cases of acute infection or disease reactivation [18].

Studies investigating *T. gondii* prevalence among blood donors in Brazil remain scarce and are concentrated in other regions with distinct climatic conditions [8,19,20]. This study is the first to determine seroprevalence and assess transfusion risk of *T. gondii* among blood donors in the Brazilian Semiarid, providing evidence to support the development of control measures aimed at minimizing the risk of toxoplasmosis in immunocompromised transfusion recipients. Considering the high seroprevalence reported in the Brazilian Semiarid and the environmental conditions that may favor parasite transmission, we hypothesized that blood donors from this region would present high exposure to *T. gondii*, with epidemiological factors associated with seropositivity, representing a potential risk for transfusion transmission. Therefore, this study aimed to determine the prevalence and risk factors associated with *T. gondii* infection among healthy blood donors eligible for donation at blood centers located in the Semiarid region of the states of Ceará and Paraíba, Northeastern Brazil, and to evaluate the risk of transfusion transmission.

## 2. Materials and Methods

### 2.1. Sampling

To determine the number of samples to be evaluated, simple random sampling was performed [21].

$$N=\frac{Z^{2}.\;p\;\left(1-p\right)}{d^{2}}$$

where

*N* = number of samples;

*Z* = standard normal distribution value corresponding to a 95% confidence level;

*p* = expected prevalence of 50%;

*d* = sampling error of 10%.

The minimum number of samples to be evaluated, as determined by the sample size calculation, was 96 blood donor samples per blood center, totaling 576 samples. However, due to the high participation rate among blood donors, 646 samples were ultimately collected.

Included were blood donors aged 18 to 69 years who met the eligibility criteria for blood donation in Brazil [15], namely: body weight of at least 50 kg, a minimum of six hours of sleep within the previous 24 h, adequate food intake, and eligibility for donation according to the clinical screening procedures of the participating blood centers. Donors who did not meet these criteria were excluded from the study. Sample collection was carried out between August and December 2025.

### 2.2. Study Design and Population

**Figure 1:**
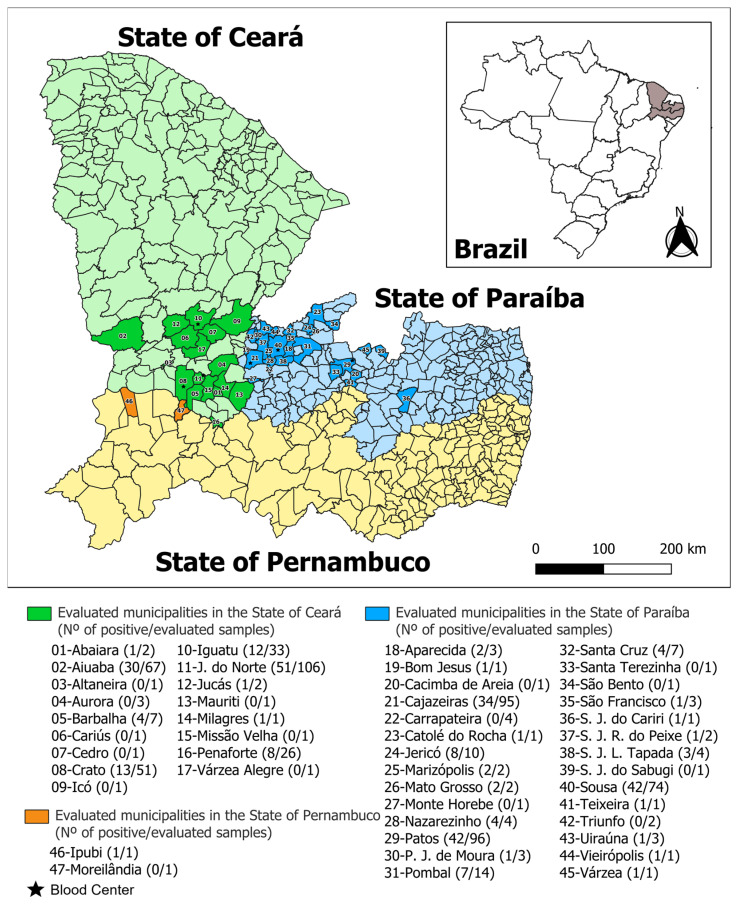
Geographical distribution of municipalities of origin, total number of samples collected, and seropositivity for anti-*T. gondii* IgG antibodies among blood donors participating in the study at blood centers located in the Semiarid region of the states of Ceará and Paraíba, Northeastern Brazil.

This was an observational, cross-sectional, and analytical study. Blood samples were collected from 646 donors in the states of Ceará and Paraíba. In Ceará, samples were obtained at the Hematology and Hemotherapy Center of Ceará (HEMOCE) (309 samples), including the blood centers of Crato (103 samples), Iguatu (105 samples) and Juazeiro do Norte (101 samples). In Paraíba, samples were collected at the Hematology and Hemotherapy Center of Paraíba (Hemocentro da Paraíba) (337 samples), including the blood centers of Cajazeiras (106 samples), Patos (103 samples) and Sousa (128 samples). Donors originated from 17 municipalities in the state of Ceará, 28 in the state of Paraíba, and two in the state of Pernambuco (Figure 1).

### 2.3. Blood Sample Collection from Donors

All volunteers who agreed to participate in this study signed an Informed Consent Form. Samples were collected in BD Vacutainer^®^ tubes (Becton Dickinson, Franklin Lakes, NJ, USA) containing clot activator gel for serological diagnosis and EDTA anticoagulant for molecular diagnosis. The tubes were connected to a sterile diversion system attached to the blood bag, known as a satellite bag or mother–child collection system, at the time of routine laboratory screening performed at the blood centers.

### 2.4. Sample Processing

Blood samples were centrifuged at 2500 rpm for 10 min, and 200 µL of the buffy coat were collected from EDTA tubes, transferred to sterile 1.5 mL microtubes free of DNases, RNases, pyrogens, dyes, and heavy metals, and stored at −20 °C until DNA extraction. Additionally, 1.5 mL of serum were obtained from clot activator gel tubes, transferred to serology microtubes, and stored at −20 °C until ELISA testing.

DNA was extracted using the Blood & Tissue DNA Mini Kit—100 Preps (Ludwig Biotecnologia^®^, Alvorada, RS, Brazil), following the manufacturer’s instructions. DNA and RNA were quantified using a microvolume spectrophotometer (L-Quant III, Loccus, Cotia, SP, Brazil) to assess extraction efficiency. DNA concentration ranged from 50 to 92 ng/µL. A total of 50 µL of extracted DNA were stored in sterile 1.5 mL microtubes and maintained at −20 °C until PCR analysis.

### 2.5. Detection of Anti-T. gondii IgM and IgG Antibodies

Anti-*T. gondii* antibodies were detected by enzyme-linked immunosorbent assay (ELISA) using the commercial BIOLISA TOXOPLASMOSE IgM and BIOLISA TOXOPLASMOSE IgG kits (Bioclin^®^, Belo Horizonte, Brazil), according to the manufacturer’s instructions. The BIOLISA TOXOPLASMOSE IgM kit has a clinical sensitivity of >99.9% and a clinical specificity of 97%, whereas the BIOLISA TOXOPLASMOSE IgG kit has a clinical sensitivity of 98.70% and a clinical specificity of 98.38%. Reactions were read using a microplate ELISA reader (LMR-96i, Loccus, Cotia, SP, Brazil).

For the BIOLISA TOXOPLASMOSE IgM assay, samples were considered positive when the index was ≥1.5, negative when ≤0.9, and indeterminate when between 0.9 and 1.5; indeterminate samples were retested to confirm the result. For the BIOLISA TOXOPLASMOSE IgG assay, samples were considered positive when the index was ≥1.0, negative when ≤0.90, and indeterminate when between 0.90 and 0.99; indeterminate samples were also retested to confirm the result.

### 2.6. Polymerase Chain Reaction (PCR)

Conventional PCR targeting a 529-bp noncoding repetitive fragment, present in approximately 200–300 copies in the *T. gondii* genome, was performed using primers TOX4 (CGCTGCAGGGAGGAAGACGAAAGTTG) and TOX5 (CGCTGCAGACACAGTGCATCTGGATT) under the following conditions: an initial incubation at 94 °C for 7 min, followed by 35 cycles of denaturation at 94 °C for 1 min, annealing at 55 °C for 1 min, and extension at 72 °C for 1 min, with a final extension at 72 °C for 10 min [22]. For a final volume of 25 µL, 1 µL of DNA was added to 9 µL of NZYTaq II 2× Green Master Mix (NZYTech, Lisbon, Portugal), 14 µL of ultrapure water, and 0.5 µL of each primer (10 µM).

PCR products were subjected to electrophoresis on 1.5% agarose gel at 100 V for 40 min and visualized under UV light. Positive samples were expected to amplify a 529-bp fragment [22]. Amplicon size of the positive control was confirmed using a 100-bp DNA ladder (GeneRuler DNA Ladder Mix™, Invitrogen^®^, Carlsbad, CA, USA). Positive and negative controls were included in all reactions.

### 2.7. Epidemiological Questionnaire

A structured epidemiological questionnaire was administered to each donor, covering sociodemographic variables (sex, age, education, family income, housing type), dietary and hygiene habits (consumption and washing of vegetables, undercooked meat, raw milk, water treatment, hand hygiene), transfusion history, prior knowledge of toxoplasmosis, contact with felids (ownership, handling, habits), gardening practices, soil exposure, animal husbandry, and history of pre-existing diseases or spontaneous abortion.

### 2.8. Data Analysis

To identify factors associated with anti-*T. gondii* IgG seropositivity, questionnaire data were analyzed in two stages: univariate and multivariate. In the univariate analysis, each independent variable was correlated with seropositivity using the chi-square test [23], and variables with *p* ≤ 0.05 were included in multivariate analysis using multiple logistic regression [24], adopting a 5% significance level. Collinearity was assessed by correlation testing. When the correlation coefficient exceeded 0.9, one variable was excluded based on biological plausibility. Model fit was evaluated using chi-square parameters and the Omnibus test. Analyses were performed using GraphPad Prism 9.0.2 software.

## 3. Results

### 3.1. Prevalence of T. gondii in Blood Donors from the States of Ceará and Paraíba, in the Brazilian Semiarid Region

Among the evaluated samples, a seroprevalence of 43.4% (281/646; 95% CI: 39.6–47.2%) for anti-*T. gondii* IgG antibodies was observed in the assessed blood donors. For anti-*T. gondii* IgM antibodies, a prevalence of 0.3% (2/646; 95% CI: 0.04–1.12%) was detected, with both samples being simultaneously positive for IgG antibodies.

In the molecular diagnosis, no sample tested positive for *T. gondii*. Therefore, parasitemia was not detected in the IgM-positive samples. All results are presented in Table 1.

### 3.2. Risk Factors Associated with the Seroprevalence of Anti-T. gondii IgG Antibodies in Blood Donors from the States of Ceará and Paraíba, in the Brazilian Semiarid Region

In the univariate analysis, the following variables were statistically significant (*p* ≤ 0.05): age (*p* < 0.0001), family income (*p* = 0.0168), educational level (*p* < 0.0001), salad washing practices (*p* < 0.0001), water source (*p* < 0.0001), consumption of raw milk (*p* < 0.0001), and duration as a blood donor (*p* < 0.0001) (Table 2).

When subjected to multivariate analysis, only the variables “age” and “salad washing practices” remained statistically significant (Table 3). Age was identified as a risk factor for *T. gondii* infection (OR = 2.26; 95% CI: 1.86–2.76), whereas salad washing (OR = 0.42; 95% CI: 0.27–0.62) exhibited a protective effect.

## 4. Discussion

This was the first study to determine the prevalence and risk factors associated with *T. gondii* infection among blood donors in the Brazilian Semiarid region. The 43.4% (281/646) prevalence of anti-*T. gondii* IgG antibodies identified in this study corroborates findings from other investigations conducted with blood donors in Brazil, particularly in the Southeast Region, where IgG seroprevalence rates of 44.7% and 48% have been reported in the state of São Paulo [20,25], and in the Southern Region, where a seroprevalence of 43.7% was reported in the states of Rio Grande do Sul and Santa Catarina [8]. These findings demonstrate that, despite climatic differences among Brazilian regions, exposure to *T. gondii* among blood donors remains high, possibly due to the interaction between environmental factors, sanitary conditions, and dietary habits.

In contrast, the findings of this study differ from those of another investigation conducted with donors in the Northeastern Region, but in a humid tropical climate area, where a 75% seroprevalence of anti-*T. gondii* IgG antibodies was reported [19]. The viability and persistence of oocysts are strongly associated with environmental factors such as humidity, temperature, vegetation, and soil characteristics [26]. According to Silva et al. [3], under semiarid conditions, characterized by high temperatures, prolonged drought periods, and low humidity, like the region where this study was conducted, oocysts may be inactivated within up to 36 h, even in soil, thereby reducing the likelihood of infection, particularly during dry periods. In humid tropical regions, the greater environmental persistence of oocysts, associated with increased exposure through contaminated water and food, may contribute to higher infection rates.

Only 0.3% (2/646) of the samples were positive for anti-*T. gondii* IgM antibodies. Low seroprevalence rates of anti-*T. gondii* IgM have been reported in different studies conducted in Brazil, with positivity rates of 1.6% [8] and 1.9% [20]. These values are comparable to those observed in studies involving blood donors from other countries, such as China (0.3%) [27], Iran (1.4%) [28], and Tunisia (0.4%) [29], reinforcing that low frequencies of recent *T. gondii* infection among blood donors appear to be consistently observed across distinct epidemiological settings. This finding may be attributed to health regulations, such as those established by the Brazilian Ministry of Health, which require the exclusion during clinical screening of donors presenting with fever or lymphadenopathy prior to donation [15]. The presence of clinical signs suggestive of the acute phase may lead to the exclusion of symptomatic candidates, potentially contributing to the lower frequency of IgM-positive results in epidemiological studies involving blood donors.

It was observed that the two samples positive for anti-*T. gondii* IgM antibodies were simultaneously positive for anti-*T. gondii* IgG antibodies. IgM antibodies are generally produced within one week after infection, rapidly increase in concentration, and subsequently decline, disappearing at highly variable rates [30]; however, they may persist for months or even years after infection, which can complicate interpretation, as their presence does not necessarily reflect ongoing parasitemia [31]. IgG antibodies appear one to three weeks after the onset of IgM production [30] and overlap between these immunoglobulins may occur during the transition from acute to chronic infection. For these reasons, positive IgM serology does not necessarily indicate active *T. gondii* infection, and subsequent molecular identification is required for confirmation [28]. Therefore, isolated serological diagnostic methods have limitations in excluding blood donation candidates with respect to *T. gondii*, and the exclusive use of ELISA tests in routine blood bank screening may result in unnecessary disposal of blood units and exclusion of potentially eligible donors.

No samples were PCR-positive, including those that tested positive for IgM. This finding is consistent with the results reported by Paraboni et al. [8], who also did not detect molecular positivity in IgM+/IgG+ samples. The persistence of IgM antibodies, traditionally associated with recent infection, which may remain detectable in the bloodstream for prolonged periods, together with the lower sensitivity of PCR in blood compared to other tissues, limits the accurate detection of *T. gondii* [32]. Molecular methods show high sensitivity for detecting *T. gondii* during parasitemic phases. However, assessment of transfusion risk depends on identifying the short period during which tachyzoites circulate in the bloodstream before tissue encystment occurs [28]. The low prevalence of anti-*T. gondii* IgM antibodies and the absence of PCR positivity suggest a low likelihood of active parasitemia and transfusion transmission at the time of donation. Nevertheless, the possibility of transmission cannot be completely excluded, particularly among immunocompromised recipients, due to the potential occurrence of low and intermittent parasitemia. Although the high cost and operational effort required to perform these tests may outweigh the benefits of their routine use in general donor screening [28], the possibility of transmitting opportunistic *T. gondii* infections from asymptomatic donors to immunocompromised recipients [33] justifies the implementation of targeted laboratory screening, combining serological tests with additional diagnostic methods, such as PCR or IgG avidity testing for *T. gondii*, in blood units intended for immunocompromised patients, aiming to prevent severe cases of toxoplasmosis in these recipients.

In the univariate analysis, the variables age, family income, educational level, salad washing practices, water source, consumption of raw milk, and duration as a blood donor were statistically significant, indicating that sociodemographic and behavioral factors may influence the likelihood of exposure to *T. gondii*. The association with age likely reflects cumulative lifetime exposure to *T. gondii* through prolonged contact with contaminated food, water, and environmental oocysts [34]. Age has previously been reported as a risk factor for *T. gondii* infection among blood donors and in the general population in Brazil [2,8,20]. Duration as a donor may reflect an older age profile or greater cumulative exposure among long-term donors. However, in contrast to the present findings, Gao et al. [27] suggested that repeated blood donations were negatively associated with *T. gondii* infection, possibly because regular donors tend to adopt healthier lifestyles and health-related behaviors compared to first-time donors.

Previous studies have also identified socioeconomic variables as risk factors for *T. gondii* infection, such as family income and educational level, indicating that lower income and education levels may be associated with poorer sanitary conditions, limited access to treated water, and reduced knowledge of preventive measures, thereby increasing exposure to the parasite [35]. Regarding behavioral and environmental factors, inadequate washing of salads, the source of drinking water, and the consumption of raw milk are associated with important transmission routes for the parasite, since *T. gondii* infection may be acquired through the ingestion of oocysts present in contaminated water and food or tachyzoites present in unpasteurized milk [36,37]. This is consistent with previous reports in Brazil and worldwide, reinforcing the role of foodborne transmission of *T. gondii* [38,39]. Inadequate sanitation conditions and limited access to treated water may further increase environmental contamination and human exposure to the parasite, particularly in endemic regions.

Although these variables were significant in the univariate analysis, caution is warranted when interpreting them, as this type of analysis does not control potential confounding factors. Thus, multivariate analysis allowed the identification of factors independently associated with *T. gondii* infection, with only the variables “age” and “salad washing practices” remaining statistically significant. Age showed a significant positive association, indicating that older donors had significantly higher odds of infection (OR = 2.26; 95% CI: 1.86–2.76), likely due to cumulative exposure to the agent. In contrast, adequate salad washing remained an independent protective factor (OR = 0.42; 95% CI: 0.27–0.62), reducing the likelihood of infection, which is related to the interruption of the *T. gondii* life cycle.

Although age remained independently associated with *T. gondii* infection in the multivariate model, this finding should be interpreted with caution. Age may not only represent cumulative exposure to the parasite over time but may also act as a proxy for other factors that were not fully captured in the final model, such as socioeconomic conditions, educational level, lifestyle habits, and food hygiene practices accumulated throughout life. Therefore, the observed association may reflect both biological and behavioral determinants of exposure. As limitations of the study, the cross-sectional design should be highlighted, as it may not allow the establishment of causal relationships between the evaluated factors and *T. gondii* infection. In addition, epidemiological information was obtained through a self-reported questionnaire, which may be subject to recall bias. Although PCR was performed using buffy coat samples, it may have limited sensitivity for detecting low or intermittent parasitemia, especially in chronically infected individuals. Finally, the results should be interpreted considering that the evaluated population consisted of eligible donors from selected blood centers in the Brazilian Semiarid region, which may limit the generalizability of the findings to other populations or regions.

## 5. Conclusions

Based on the findings of the present study, it can be concluded that blood donors in the Semiarid region of the States of Ceará and Paraíba, Northeast Brazil, exhibit a substantial prevalence of anti-*T. gondii* IgG antibodies. The low seroprevalence of anti-*T. gondii* IgM antibodies, which were simultaneously positive for IgG, together with the absence of molecular detection of the agent by PCR, suggests a low likelihood of active transfusion transmission of *T. gondii* in the Brazilian Semiarid region, although the possibility of transmission cannot be completely excluded, particularly among immunocompromised recipients.

Age was identified as a risk factor for seropositivity, significantly increasing the odds of infection, suggesting that older donors experience cumulative exposure to *T. gondii* throughout life. In contrast, adequate washing of salads acts as a protective factor, reducing the likelihood of *T. gondii* infection.

## Figures and Tables

**Table 1:** Distribution of samples positive for anti-*T. gondii* IgM and IgG antibodies and conventional PCR among blood donors from the Hematology and Hemotherapy Centers of the States of Ceará and Paraíba, Northeast Brazil.

State of Ceará	Number of Samples Collected	Positives ELISA IgM (%)	Positives ELISA IgG (%)	Positives PCR (%)
Blood Center of Crato	103	0 (0)	34 (33)	0 (0)
Blood Center of Iguatu	105	1 (0.9)	43 (40.9)	0 (0)
Blood Center of Juazeiro do Norte	101	0 (0)	46 (45.5)	0 (0)
Total of Ceará State	309	1 (0.3)	123 (39.8)	0 (0)
State of Paraíba				
Blood Center of Cajazeiras	106	1 (0.9)	39 (36.7)	0 (0)
Blood Center of Patos	103	0 (0)	43 (41.7)	0 (0)
Blood Center of Sousa	128	0 (0)	76 (59.3)	0 (0)
Total of Paraíba State	337	1 (0.3)	158 (46.8)	0 (0)
Total	646	2 (0.3)	281 (43.4)	0 (0)

**Table 2:** Univariate analysis of the association between the presence of anti-*T. gondii* IgG antibodies and risk factors for infection in blood donors from the Hematology and Hemotherapy Centers of the States of Ceará and Paraíba, Northeast Brazil.

Variable	Category	Number of Participants	Positives (%)	*p*-Value
Age	18–29 years	229	51 (22.2)	<0.0001 *
	30–41 years	211	89 (42.1)	
	42–53 years	135	92 (68.1)	
	54–65 years	52	43 (82.6)	
	>65 years	9	6 (66.6)	
Family income	Up to 1 minimum wage	265	128 (47.1)	0.0168 *
	1–2 minimum wages	203	88 (43.3)	
	3–4 minimum wages	106	32 (30.1)	
	4–5 minimum wages	29	14 (48.2)	
	>5 minimum wages	32	16 (50)	
Education level	Illiterate	18	16 (88.9)	<0.0001 *
	Incomplete elementary school	98	56 (57.1)	
	Complete elementary school	30	19 (63.3)	
	Incomplete high school	54	26 (48.1)	
	Complete high school	212	81 (38.2)	
	Incomplete higher education	94	30 (31.9)	
	Complete higher education	140	52 (37.1)	
Salad washing practices	Yes	531	247 (46.5)	<0.0001 *
	No	86	33 (38.3)	
	Not applicable	29	0 (0)	
Water source	Tap water	193	85 (44)	<0.0001 *
	Bottled mineral water	330	131 (39.7)	
	Artesian well	28	14 (50)	
	Reservoir	4	3 (75)	
	Hand-dug well	5	3 (60)	
	Rainwater	17	15 (88.2)	
	Cistern	33	15 (45.5)	
	Water truck	252	19 (7.5)	
	Does not know	7	4 (57.1)	
Raw milk consumption	Yes	82	34 (41.4)	<0.0001 *
	No	280	246 (87.8)	
	Up to 1 year	182	52 (28.6)	
Duration as a blood donor	2–4 years	143	57 (39.9)	<0.0001 *
	>5 years	299	162 (54.2)	
	Does not know	22	9 (40.9)	

*: statistically significant.

**Table 3:** Multivariate analysis of the association between the presence of anti-*T. gondii* IgG antibodies and risk factors for infection in blood donors from the Hematology and Hemotherapy Centers of the States of Ceará and Paraíba, Northeast Brazil.

Variable	Odds Ratio (95% IC)	*p*-Value	Significance
Age	2.26 (1.86–2.76)	<0.0001	*
Family income	0.92 (0.78–1.07)	0.3080	ns
Educational level	0.91 (0.82–1.02)	0.1431	ns
Salad washing practices	0.42 (0.27–0.62)	<0.0001	*
Water source	0.97 (0.89–1.06)	0.6217	ns
Raw milk consumption	1.15 (0.69–1.94)	0.5730	ns
Duration as a blood donor	1.11 (0.59–2.11)	0.7303	ns

ns: not significant; *: statistically significant.

## Data Availability

The original contributions presented in this study are included in the article. For further information, please contact the corresponding author.

## Abbreviations

The following abbreviations are used in this manuscript:

CNPq	National Council for Scientific and Technological Development
DNA	Deoxyribonucleic acid
EDTA	Ethylenediaminetetraacetic acid
ELISA	Enzyme-Linked Immunosorbent Assay
HEMOCE	Center of Hematology and Hemotherapy of Ceará
IC95%	95% Confidence Interval
IFPB	Federal Institute of Paraíba
IgG	Immunoglobulin G
IgM	Immunoglobulin M
INCT	National Institute of Science and Technology
OR	Odds Ratio
PCR	Polymerase Chain Reaction
*T. gondii*	*Toxoplasma gondii*

## References

[1] Dubey J.P. (2021). Toxoplasmosis of Animals and Humans.

[2] Aloise D.A., Coura-Vital W., Carneiro M., Rodrigues M.V., Toscano G.A.C., Silva R.B., Andrade Neto V.F., Vitor R.W.A. (2018). Seroprevalence and risk factors for human toxoplasmosis in Northeastern Brazil. J. Trop. Pathol..

[3] Silva A.L.P., Lima B.A., Formiga V.H.A.S., Lima E.F., Silva Filho G.M., Silva W.I., Silva J.O., Alvares F.B.V., Vilela V.L.R., Feitosa T.F. (2025). Survival and viability of *Toxoplasma gondii* oocysts under natural dry season conditions in the Brazilian semi-arid region. Vet. Res. Commun..

[4] Vilela V.L.R., Feitosa T.F., Simões S.V.D., Mota R.A., Katzer F., Bartley P.M. (2023). An abortion storm in a goat farm in the Northeast Region of Brazil was caused by the atypical *Toxoplasma gondii* genotype #13. Curr. Res. Parasitol. Vector-Borne Dis..

[5] Vilela V.L.R., Feitosa T.F. (2024). Recent advances in *Toxoplasma gondii* infection and toxoplasmosis. Trop. Med. Infect. Dis..

[6] Silva Filho G.M., Silva J.O., Costa Filho A.A., Parentoni R.N., Brasil A.W.L., Feitosa T.F., Vilela V.L.R. (2025). Seroprevalence of anti-*Neospora caninum* and anti-*Toxoplasma gondii* antibodies in cattle intended for human consumption in the State of Paraíba, Brazil. Ruminants.

[7] Almeria S., Dubey J.P. (2021). Foodborne transmission of *Toxoplasma gondii* infection 169 in the last decade. An overview. Res. Vet. Sci..

[8] Paraboni M.L.R., Commodaro A.G., Campi-Azevedo A.C., Brito-de-Sousa J.P., Gonçalves I.L., da Costa D.F., Ribeiro K.S., Garcia J.L., Silveira C., Martins-Filho O.A. (2022). Seroprevalence and systemic immune biomarkers associated with *Toxoplasma gondii* infection in blood donors from Southern Brazil. Immunobiology.

[9] Siegel S.E., Lunde M.N., Gelderman A.H., Halterman R.H., Brown J.A., Levine A.S., Graw R.G. (1971). Transmission of toxoplasmosis by leukocyte transfusion. Blood.

[10] Zhou N., Fu H., Wang Z., Shi H., Yu Y., Qu T., Wang L., Zhang X., Wang L. (2019). Seroprevalence and risk factors of *Toxoplasma gondii* infection in children with leukemia in Shandong Province, Eastern China: A case-control prospective study. PeerJ.

[11] Bessa G.L., Vitor R.W.A., Martins-Duarte E.S. (2021). *Toxoplasma gondii* in South America: A differentiated pattern of spread, population structure and clinical manifestations. Parasitol. Res..

[12] Khurana S., Batra N. (2016). Toxoplasmosis in organ transplant recipients: Evaluation, implication, and prevention. Trop. Parasitol..

[13] Dubey J.P., Murata F.H.A., Cerqueira-Cézar C.K., Kwok O.C.H., Villena I. (2021). Congenital toxoplasmosis in humans: An update of worldwide rate of congenital infections. Parasitology.

[14] Foroutan-Rad M., Majidiani H., Dalvand S., Daryani A., Kooti W., Saki J., Hedayati-Rad F., Ahmadpour E. (2016). Toxoplasmosis in Blood Donors: A Systematic Review and Meta-Analysis. Transfus. Med. Rev..

[15] Portaria nº 158, de 4 de Fevereiro de 2016. https://bvsms.saude.gov.br/bvs/saudelegis/gm/2016/prt0158_04_02_2016.html.

[16] Fortes M.S., Lopes-Mori F.M.R., Caldart E.T., Constantino C., Evers F., Pagliari S., de Almeida J.C., Barros L.D., Freire R.L., Garcia J.L. (2018). Caprine toxoplasmosis in Southern Brazil: A comparative seroepidemiological study between the indirect immunofluorescence assay, the enzyme-linked immunosorbent assay, and the modified agglutination test. Trop. Anim. Health Prod..

[17] Brenier-Pinchart M.P., Filisetti D., Cassaing S., Varlet-Marie E., Robert-Gangneux F., Delhaes L., Guitard J., Yéra H., Bastien P., Pelloux H. (2022). Molecular diagnosis of toxoplasmosis: Multicenter evaluation of the *Toxoplasma* RealCycler Universal PCR assay on 168 characterized human samples. J. Mol. Diagn..

[18] Brenier-Pinchart M.P., Capderou E., Bertini R.L., Bailly S., Fricker-Hidalgo H., Varlet-Marie E., Murat J.B., Sterkers Y., Touafek F., Bastien P. (2015). Molecular diagnosis of toxoplasmosis: Value of the buffy coat for the detection of circulating *Toxoplasma gondii*. Diagn. Microbiol. Infect. Dis..

[19] Coêlho R.A., Kobayashi M., Carvalho L.B. (2003). Prevalence of IgG antibodies specific to *Toxoplasma gondii* among blood donors in Recife, Northeast Brazil. Rev. Inst. Med. Trop. Sao Paulo.

[20] Assoni L.C.P., Nakashima F., de Sousa V.P., Paduan N.J., Andreasse I.R., Anghinoni T.H., Faria Junior G.M., Ricci Junior O., Castiglioni L., Brandão C.C. (2024). Seroepidemiology of *Toxoplasma gondii* infection in blood donors in a population from the northwestern region of São Paulo State, Brazil. Trans. R Soc. Trop. Med. Hyg..

[21] Thrusfield M.V. (2018). Veterinary Epidemiology.

[22] Homan W.L., Vercammen M., Braekeleer J., Verschueren H. (2000). Identification of a 200- to 300-fold repetitive 529 bp DNA fragment in *Toxoplasma gondii*, and its use for diagnostic and quantitative PCR. Int. J. Parasitol..

[23] Zar J.H. (1999). Biostatistical Analysis.

[24] Hosmer D.W., Lemeshow S. (2000). Applied Logistic Regression.

[25] Nakashima F., Pardo V.S., Miola M.P., Murata F.H.A., Paduan N., Longo S.M., Brandão de Mattos C.C., Pereira-Chioccola V.L., Ricci O., de Mattos L.C. (2020). Serum IgG anti-*Toxoplasma gondii* antibody concentrations do not correlate nested PCR results in blood donors. Front. Cell. Infect. Microbiol..

[26] Shapiro K., Bahia-Oliveira L., Dixon B., Dumètre A., de Wit L.A., VanWormer E., Villena I. (2019). Environmental transmission of *Toxoplasma gondii*: Oocysts in water, soil and food. Food Waterborne Parasitol..

[27] Gao J., Zhang J., Chen H., Ruan W., Feng Y., Lu Q., Wang X., Jiang J. (2025). Prevalence and risk factors associated with *Toxoplasma gondii* infection among blood donors in eastern China. BMC Infect. Dis..

[28] Foroutan M., Majidiani H., Hassanipour S., Badri M. (2024). *Toxoplasma gondii* seroprevalence in Iranian blood donors: A systematic review and meta-analysis. Heliyon.

[29] Lachkhem A., Lahmar I., Galal L., Babba O., Mezhoud H., Hassine M., Lachkhem A., Dardé M.L., Mercier A., Babba H. (2020). Seroprevalence of *Toxoplasma gondii* among healthy blood donors in two locations in Tunisia and associated risk factors. Parasite.

[30] Teimouri A., Mohtasebi S., Kazemirad E., Keshavarz H. (2020). Role of *Toxoplasma gondii* IgG avidity testing in discriminating between acute and chronic toxoplasmosis in pregnancy. J. Clin. Microbiol..

[31] Vargas-Villavicencio J.A., Cedillo-Peláez C., Aguilar-Orozco M.I., Rico-Torres C.P., Farfan-Morales J.E., Correa D. (2022). Vertical transmission and pathological findings in the mother, the placenta and the offspring, during first and last thirds of gestation in a mouse model of congenital toxoplasmosis. Parasitol. Int..

[32] Kantzanou M., Kostares E., Kostare G., Papagiannopoulou E., Kostares M., Tsakris A. (2025). Prevalence of *Toxoplasma gondii* infection among blood donors: A systematic review and meta-analysis. Cureus.

[33] Baloochi M., Amini Fard M.R., Ordoni R., Pendar A., Vafae Eslahi A., Badri M., Dezhkam A., Kalkali M., Rahdar H.A., Mohammadi M. (2025). Seroepidemiology of *Toxoplasma gondii* infection among patients with beta-thalassemia major: A case-control study in Southeastern Iran. BMC Infect. Dis..

[34] Lupu M.A., Lighezan R., Paduraru A.A., Dragomir A., Pavel R., Grada S., Mihu A.G., Ursoniu S., Olariu T.R. (2022). Seroepidemiology of *Toxoplasma gondii* infection in blood donors from Western Romania. Microorganisms.

[35] Mareze M., Benitez A.D.N., Brandão A.P.D., Pinto-Ferreira F., Miura A.C., Martins F.D.C., Caldart E.T., Biondo A.W., Freire R.L., Mitsuka-Breganó R. (2019). Socioeconomic vulnerability associated with *Toxoplasma gondii* exposure in southern Brazil. PLoS ONE.

[36] Akbari M., Azadi D., Habibi D., Khodashenas S., Shariatmadari F., Abedi B. (2022). Toxoplasmosis infection in newborns: A systematic review and meta-analysis. Adv. Biomed. Res..

[37] Khan M.Y., Barlaam A., Ferrari N., Gazzonis A.L., Normanno G.G., Jiménez-Meléndez A., Robertson L.J., Giangaspero A. (2025). Molecular detection and genetic characterization of *Toxoplasma gondii* in goats’ milk and aborted goat kid tissues from Pakistan. Food Waterborne Parasitol..

[38] López Ureña N.M., Chaudhry U., Calero Bernal R., Cano Alsua S., Messina D., Evangelista F., Betson M., Lalle M., Jokelainen P., Ortega Mora L.M. (2022). Contamination of soil, water, fresh produce, and bivalve mollusks with *Toxoplasma gondii* oocysts: A systematic review. Microorganisms.

[39] Walana W., Odai S.A., Tamomh A.G. (2026). Prevalence, risk factors, diagnosis and outcomes of *Toxoplasma gondii* infection in pregnancy: A review. Parasitol. Int..

